# Retraction Note: Fe_3_O_4_@void@C-Schiff-base/Pd yolk-shell nanostructures as an effective and reusable nanocatalyst for Suzuki coupling reaction

**DOI:** 10.1038/s41598-025-20023-8

**Published:** 2025-09-15

**Authors:** Aliyeh Barzkar, Alireza Salimi Beni, Shahab Parang, Farhang Salahshour

**Affiliations:** 1 Department of Chemistry, Faculty of Science, Yasouj University, Yasouj, 75918-74831 Iran; 2 Department of Chemistry, Faculty of Science, Vali-E-Asr University, P.O. Box 77176, Rafsanjan, Islamic Republic of Iran; 3 Department of Pharmaceutics, School of Pharmacy, Shiraz University of Medical Sciences, Shiraz, Iran

Retraction of: *Scientific Reports* 10.1038/s41598-023-46839-w, published online 15 November 2023

The Editors have retracted this Article.

After publication, concerns were raised about the validity of the traces in Figure 3. Upon further investigation, the data was found to be reused in a previously published Article by different Authors, partially describing different conditions^1^. The data provided by Authors did not sufficiently address the concerns raised. The Editor has lost confidence in the data and conclusions of this Article.

Farhang Salahshour, Alireza Salimi, Shahab Parang and Aliye Barzekar disagree with this retraction.
